# School-Class Co-Ethnic and Immigrant Density and Current Smoking among Immigrant Adolescents

**DOI:** 10.3390/ijerph17020598

**Published:** 2020-01-17

**Authors:** Matthias Robert Kern, Andreas Heinz, Helmut Erich Willems

**Affiliations:** Department of Social Sciences, University of Luxembourg, 4366 Esch-sur-Alzette, Luxembourg; andreas.heinz@uni.lu (A.H.); helmut.willems@uni.lu (H.E.W.)

**Keywords:** migration, smoking, substance use, adolescence, ethnic density, ethnic composition, school-class, multilevel analysis, HBSC, acculturative stress

## Abstract

Although the school-class is known to be an important setting for adolescent risk behavior, little is known about how the ethnic composition of a school-class impacts substance use among pupils with a migration background. Moreover, the few existing studies do not distinguish between co-ethnic density (i.e., the share of immigrants belonging to one’s own ethnic group) and immigrant density (the share of all immigrants). This is all the more surprising since a high co-ethnic density can be expected to protect against substance use by increasing levels of social support and decreasing acculturative stress, whereas a high immigrant density can be expected to do the opposite by facilitating inter-ethnic conflict and identity threat. This study analyses how co-ethnic density and immigrant density are correlated with smoking among pupils of Portuguese origin in Luxembourg. A multi-level analysis is used to analyze data from the Luxembourg Health Behavior in School-Aged Children study (*N* = 4268 pupils from 283 classes). High levels of co-ethnic density reduced current smoking. In contrast, high levels of immigrant density increased it. Thus, in research on the health of migrants, the distinction between co-ethnic density and immigrant density should be taken into account, as both may have opposite effects.

## 1. Introduction

Adolescence is a time of heightened risk taking when substance use is usually initiated [1]. As peer evaluations gain utmost importance during this period [2] peer modelling and peer pressure mechanisms unfolding in the school-class setting play a central role in determining adolescent substance use [3]. In the case of immigrant adolescents the role of the school-class and its ethnic composition might be even more pronounced because school-classes constitute one of the most important acculturation contexts [4] and, as such, their ethnic makeup is likely to affect levels of acculturative stress among them, which, in turn, can have an impact on substance use [5,6]. As school-classes in many countries increasingly vary in their ethnic composition [7], investigating the association between school-class ethnic composition and substance use among immigrant adolescents becomes increasingly important. 

Research examining the relationship between the ethnic composition of people’s social environment and their health and health behavior often yields results in favor of the so-called “ethnic density hypothesis”, which, in its most general form, posits that “as the proportion of an ethnic minority group […] increases, their health complications will decrease” [8] (p. 16). However, studies examining those effects differ in how they conceptualize ethnic density. 

While some studies investigate how the health of members of different ethnic minority groups is affected by the share of co-ethnics (i.e., members of their own ethnic group) within their immediate social environment, others are interested in how the health of immigrants in general is affected by the share of immigrants in their immediate social environment [9]. To distinguish those two concepts we will speak of co-ethnic density, to describe the relative size of a specific ethnic group in relation to the whole population and of immigrant density to describe the relative size of all immigrants in relation to the whole population [8]. While there are relatively strong theoretical reasons to assume a protective effect of co-ethnic density, the case for a protective effect of immigrant density is considerably weaker. 

Co-ethnic density might exert a protective effect on substance use among immigrant adolescents by lowering levels of acculturative stress [10,11,12] and by increasing levels of social support [9,13].

High levels of acculturative stress facilitate substance use among immigrant adolescents [5,6]. One important driver of acculturative stress is the discordance between internal and external acculturation, so the discrepancy between culturally-transmitted preferences, attitudes and feelings, and actual behavior [14]. High levels of co-ethnic density might lower this discordance by opening up opportunities to behave in accordance with one’s preferences (for instance, speaking one’s mother tongue) and, thus, reduce levels of acculturative stress [10,11,12].

Social support, on the other hand, is one of the major stress mitigating factors for immigrants [13]. Since, social networks are often based on criteria, such as ethnicity [15], levels of social support tend to grow with the number of co-ethnics within one’s social environment. 

In line with this, a small number of studies found a protective effect of neighborhood level co-ethnic density on substance use. Bécares, Nazroo, and Stafford, for instance, detected a protective effect of neighborhood co-ethnic density on current drinking among Indian and Pakistani adults in Britain [16]. Similarly, Kim and McCarthy report a lower risk of alcohol and tobacco use among school students of Asian descent if they live in neighborhoods with many other immigrants from Asia [17]. 

As mentioned before, a protective effect of immigrant density is theoretically less convincing. A large proportion of immigrants from countries of origin other than one’s own does not necessarily provide an environment in which one can behave in accordance with one’s culturally transmitted preferences or form social support networks based on cultural similarity. What is more, such an environment might even have a detrimental effect, due to increased interethnic tensions [18] and identity threat [19]. Vervoort, Scholte and Scheepers found that a higher proportion of ethnic minority students in class was associated with more negative attitudes toward ethnic minority students not only among ethnic majority students, but also among ethnic minority students themselves [18]. Accordingly, high levels of immigrant density might increase both the likelihood of racist victimization by ethnic majority students, and the likelihood of interethnic conflict between ethnic minorities. In a similar vein, Veldhuizen and colleagues found that the relative proportion of Moroccans in one’s residential area had a detrimental effect on self-assessed health among Turks living in the Netherlands [19]. They speculated this might be due to the threat that an increased presence of another minority ethnic group, perceived as similar, poses towards one’s group identity. 

Nevertheless, the few studies that investigated the effect of immigrant density on substance use generally found a protective effect. Monshouwer and colleagues found a protective effect of immigrant density on the school level on episodic heavy drinking for immigrant adolescents [20], and Jackson and colleagues found a protective effect of neighborhood immigrant concentration on alcohol use among all adolescents, irrespective of their own migration status [21].

However, these studies did not control for co-ethnic density as a potential confounder. Since high levels of immigrant density might go hand in hand with high levels of co-ethnic density at least for the largest ethnic groups, this might introduce omitted-variable bias. The observed protective effect of immigrant density might more adequately be attributed to co-ethnic density. To disentangle the effects of co-ethnic and immigrant density, it is paramount to assess both factors simultaneously, which is what we do in the current study.

In particular, we assess the net-effects of school-class co-ethnic density and school-class immigrant density on current smoking among immigrant adolescents. While adolescent smoking has declined substantially throughout the last 40 years, its highly addictive nature [22], as well as the many negative health consequences associated with it [23], still render it a major health concern [24]. Measuring co-ethnic and immigrant density on the school-class level provides a number of advantages. Adolescents attending the same school-class spent large amounts of time interacting with one another, rendering the school-class a particular relevant social environment for them. In light of the compelling role of peers in shaping adolescent substance use, school-classes offer the additional benefit of comprising adolescents within the same age range [25].

Given that both factors are theoretically distinct and can potentially exert contrasting effects we assess them simultaneously. To detect potential effects, the context in which they are studied has to offer a wide enough range in both co-ethnic- and immigrant density [8]. This is achieved in the current study by examining the case of immigrant adolescents of Portuguese descent in Luxembourg. Almost 48% of the population of Luxembourg are foreign nationals, making Luxembourg the country with the highest proportion of foreign nationals in the EU (European Union) [26]. People of Portuguese nationality make up 37% of the foreign population and 16% of the whole population of Luxembourg [27,28], making them the largest immigrant group. As a consequence of the large number of immigrants in general and of immigrants of Portuguese descent in particular in Luxembourg, immigrant adolescents of Portuguese descent in Luxembourg attend school-classes that vary considerably both in immigrant and co-ethnic density, providing us with the ideal setting to investigate those ethnic compositional effects. The first wave of Portuguese immigration to Luxembourg occurred in the 1970s as a result of the guest worker agreement between Luxembourg and Portugal, which allowed Portuguese labor migrants to bring their immediate family with them [29]. Since then, the number of people of Portuguese descent in Luxembourg has risen steadily. Due to a continued influx of Portuguese immigrants, second-generation immigrants of Portuguese descent live alongside very recent immigrants from Portugal [28]. In contrast to many immigrants from earlier immigration waves to Luxembourg, intergenerational upward social mobility is less common among immigrants from Portugal and, on average, their level of education is lower than that of native Luxembourgers or other immigrant groups [28,30]. 

In a first step, we assess the direct effect of both factors on current smoking for the subsample of adolescents of Portuguese descent (*N* = 1156). Based on our previous discussion we expect the net effect of co-ethnic density to be protective and the net effect of immigrant density to be detrimental. 

In a second step, using the full sample (*N* = 4242) we assess whether potential differences in smoking behavior between native Luxembourgish students and students of Portuguese descent differ between school-classes based on the proportion of students of Portuguese descent and the share of immigrants in class. Given that there is no reason to expect a protective effect of the share of students of Portuguese descent in class on native Luxembourgish students, we expect students of Portuguese descent to do better in relation to their Luxembourgish classmates in classes with many co-ethnics than in classes with few co-ethnics. Given that the proposed mechanisms for a detrimental effect of immigrant density do not apply in the same way to native Luxembourgers as to the students of Portuguese descent, we expect students of Portuguese descent to do worse in relation to their native classmates in classes with many immigrants than in classes with few. 

## 2. Materials and Methods 

### 2.1. Data

Data are retrieved from the 2014 wave of the Luxembourgish Health Behavior in School-aged Children (HBSC) study. The HBSC study is a large cross-national survey, aimed at investigating health and health behavior and its social determinants among children and adolescents. It follows a clustered sampling procedure where all students attending one of the randomly chosen school-classes are asked to participate [24]. Informed consent was obtained from all individual participants or their legal guardians. In Luxembourg 590 school-classes comprising 9648 students were drawn [31]. Of those, 7757 students between the ages of 9 and 19 completed the survey. We excluded 2183 students below the age of 12 or attending primary schools. Limiting our investigation to students attending secondary schools has the benefit that there is a decreased risk of confounding by neighborhood context. In Luxembourg, given the small size of the country and a lack of restrictions, pupils often chose secondary schools irrespective of their proximity to their place of residence. We, furthermore, excluded 601 students attending classes with less than ten participants because their class size was too small to aggregate reliable ethnic compositional measures and 731 students because they had missing values on one of the variables in the model. Our analyses are based on 4242 cases, spread out over 283 classes, of secondary school-students between the ages of 12 and 19 who have valid responses on all of the variables in the model (male: 1915, female: 2327). Among them are 1165 students of Portuguese descent (male: 503, female: 662) distributed over 254 classes. Within the whole sample the share of students of Portuguese descent in class ranged between 0% and 84.6% with a mean of 27%. The share of immigrants in class ranged between 0% and 100% with a mean of 62.8%. Among the subsample of students of Portuguese descent, co-ethnic density ranged from 3.6% to 84.6% with a mean of 42.7%, and immigrant density ranged from 16.7% to 100% with a mean of 74.8%. 

### 2.2. Measures

#### 2.2.1. Dependent Variable

Current smoking was assessed with an item: “How often do you smoke tobacco at present?” with response categories (1) every day, (2) at least once a week but not every day, (3) less than once a week, and (4) I do not smoke and was dichotomized so that responses 1–3 were coded as 1 and response 4 was coded as 0.

#### 2.2.2. Predictor Variables

Immigrant status and Portuguese ethnicity were assessed using variables on the country of birth of the adolescents and their parents. Adolescents were regarded as having a migration background if at least one of their parents were born outside of Luxembourg. Accordingly, both first- and second generation immigrants were regarded as having a migration background. Immigrant density is the percentage of students with a migration background in class. 

Adolescents were regarded as of Portuguese descent if they were either themselves born in Portugal or they were born in Luxembourg to at least one parent born in Portugal. Robustness tests reveal that excluding adolescents with a native-born parent from the definition does not substantively alter our results. Co-ethnic density was calculated as the percentage of students of Portuguese descent in class. To account for the fact that the aggregated co-ethnic and immigrant density might be unreliable for classes with only few participants, classes with less than 10 cases with valid responses on parents’ country of birth were excluded from further analyses [32]. Informed by the assumption that increases at higher levels of co-ethnic density and immigrant density exert weaker effects than increases at lower values, both measures were log transformed. 

#### 2.2.3. Control Variables

On the school-class level we controlled for educational track and mean age in class. 

Upon finishing primary school, school students in Luxembourg are assigned to one of two educational tracks. The track “enseignement secondaire classique” primarily aims at preparing school students for higher education, whereas the track “enseignement secondaire technique” primarily aims at preparing school students for vocational training. Within the enseignement secondaire technique students lacking in particular academic skills are assigned to the modulaire track leaving us with a total of three educational tracks [33]. Students of Portuguese descent, as well as students from a lower socio-economic background are underrepresented in the enseignement secondaire classique and overrepresented in the enseignement secondaire technique and the enseignement secondaire technique modulaire [33]. To account for a potential confounding through differences in the socio-economic and ethnic composition of the different educational tracks we included them as control variables in our models. To control for exposure to classmates of a different age we also included the mean age in class as a potential confounder. 

On the individual level we controlled for potential socio-economic and socio-demographic confounders: gender, relative age (individual age centered around the mean age in class) and familial socio-economic status. Familial socio-economic status was measured using the Family Affluence Scale (FAS III) [34]. Furthermore we controlled for proxies of acculturation: being a first generation immigrant (i.e., being born outside of Luxembourg), Portuguese language use at home (yes/no), Luxembourgish language use at home (yes/no), and risk factors of substance use: disliking school (1 = like a lot to 4 = do not like at all) and how often they spend time with their friends after 8 p.m. (1 = hardly ever or never to 4 = daily) [35].

### 2.3. Statistical Analysis

Analyses consisted of a number of multilevel logistic regressions using Stata 14’s melogit (Stata Corp., College Station, TX, USA). In a first step, we assessed the direct effects of co-ethnic density and immigrant density on substance use among students of Portuguese descent. To this end we ran a multilevel logistic regression only for the subpopulation of students of Portuguese descent (*N* = 1165).

In a second step, we assessed whether Portuguese-ethnic density and immigrant density moderate potential differences in substance use between students of Portuguese descent and native Luxembourgers. To this end, using the whole sample of adolescents between 12 and 19 years of age attending secondary schools (*N* = 4242), we ran a multilevel logistic regression including cross-level interactions between a dummy for being of Portuguese descent (with being native Luxembourgish as the reference category) and Portuguese ethnic density and immigrant density. By simultaneously including both interaction terms as well as their constituent terms in the model, it was possible to assess the moderation of the effect of being of Portuguese descent by Portuguese ethnic density after controlling for the moderation through immigrant density and the other way around [36]. In order to adjust for the clustered sampling procedure of the HBSC study [24], pseudo-likelihood estimation methods based on Taylor series linearization were used [37,38]. Consequently, since likelihood ratio tests are not applicable, significance of variance components was assessed using one-tailed adjusted Wald tests [39]. 

School-classes with high levels of co-ethnic or immigrant density might concentrate in schools with particularly high or low overall levels of current smoking. If this is the case, we might misattribute effects of unobserved school-level characteristics to school-class co-ethnic or immigrant density [40]. To examine this possibility, we assessed how much of the overall variance in current smoking is explained at the school-level by fitting a three-level null model with pupils nested in school-classes and school-classes nested in schools. We did this separately for the subsample of students of Portuguese descent and the whole sample. For the subsample of students of Portuguese descent we found that the amount of variance explained at the school-level was very low with 1.3% of the overall variance and was not statistically significantly greater than zero. Accordingly, the chances of unobserved school-level characteristics confounding our results were minimal and we decided in favor of a more parsimonious two level model with pupils nested in school-classes. Robustness tests revealed that controlling for school-level clustering either using a fixed or a random effects approach [40] did not substantively alter our results. Within the overall sample, the amount of variance explained at the school-level was slightly larger with 2.9% of the overall variance and was significantly greater than zero. Accordingly, in those cases we controlled for potential confounding by unobserved school-level characteristics by including school as a third level in our multilevel analysis. 

### 2.4. Ethical Approval

The study was approved by the Comité National d’Ethique de Recherche (CNER). Furthermore, the Commission nationale pour la protection des données (CNPD) was informed about the carrying out of the survey.

## 3. Results

### 3.1. The Effect of Co-Ethnic and Immigrant Density on Students of Portuguese Descent

A total of 20.3% of the students of Portuguese descent in our sample report being current smokers. The intraclass correlation (ICC) for the null model is 16%, indicating an amount of clustering at the class level that warrants the use of multilevel methods. As can be seen in Table 1, there is a highly significant detrimental effect of mean age in class. The corresponding odds ratio (OR) is 1.47, which means that a one year increase in mean age in class leads to a 47% increase in the odds of current smoking. Relative age, however, does not exert a significant effect. There is a significant detrimental effect of being female. Girls of Portuguese descent have 42% higher odds of being current smokers than their male counterparts. Luxembourgish language use at home has a significant detrimental effect. Adolescents of Portuguese descent who speak Luxembourgish at home have 53% higher odds of being current smokers. The educational track affects current smoking considerably. In comparison to adolescents participating in the highest track, the enseignement secondaire classique the odds for current smoking are increased by 82% for students participating in the vocationally-oriented enseignement secondaire technique, and by 170% for students participating in the enseignement secondaire technique—modulaire, which caters to those students lacking particular academic skills. Both disliking school and time spent with friends after 8 p.m. increase the likelihood of current smoking. The less adolescents of Portuguese descent like school and the more frequently they spent time with friends after 8 p.m. the more likely they are to be current smokers.

#### 3.1.1. Co-Ethnic Density

**Figure 1:**
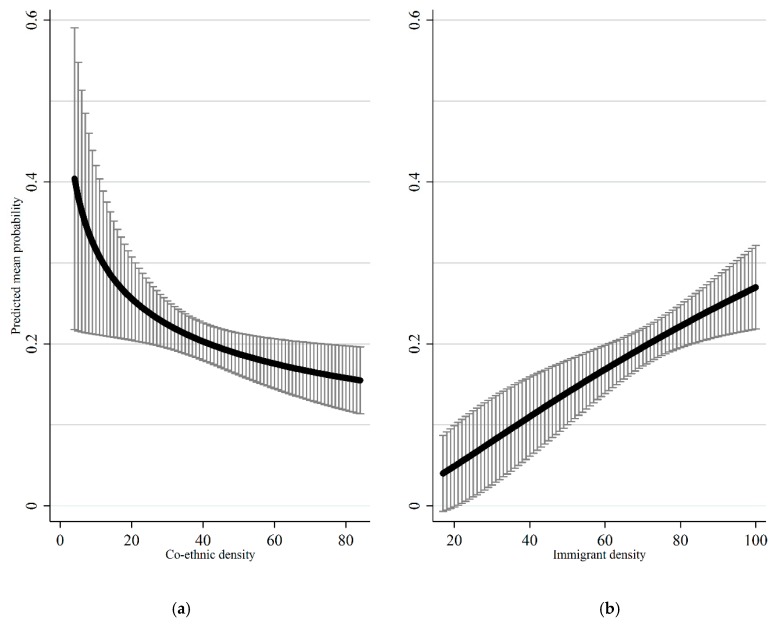
(**a**) Predicted mean probability of current smoking by co-ethnic density in school-class with 95% confidence intervals; (**b**) predicted mean probability of current smoking by immigrant density in class with 95% confidence intervals. Note: All other variables held at their observed values in the data.

As can be seen from the odds ratios in Table 1, higher levels of co-ethnic density are associated with a lower likelihood of current smoking. The OR for the log transformed variable is 0.6. Figure 1 reveals how this can be interpreted: At 4% co-ethnic density, which corresponds to the lowest level of co-ethnic density observed in the subsample, the mean predicted probability of ever having smoked is 40.4%. In contrast, at 84% co-ethnic density, which corresponds to the highest value in the subsample, this value lies at 15.5%. 

#### 3.1.2. Immigrant Density

As shown in Table 1, as immigrant density increases, the odds for lifetime occurrence of smoking increase. The OR for the log transformed variable is 3.89. In Figure 1 we see that between students of Portuguese descent attending classes with the lowest level of immigrant density and those attending classes with the highest level, the predicted mean probability increases more than six-fold from 4% at 17% immigrant density to 27% at 100% immigrant density.

To conclude, the subgroup analysis revealed that, in line with our hypotheses, among adolescents of Portuguese descent, co-ethnic density exerts a significant protective effect on current smoking after controlling for the effect of immigrant density. At the same time, there was a significant detrimental effect of immigrant density after controlling for co-ethnic density. 

### 3.2. Moderation of the Effect of Being of Portuguese Descent

For classes attended by at least one student of Portuguese descent and one native Luxembourgish student, Portuguese ethnic density ranged from 3.6% to 76.2%, the corresponding range of immigrant density was between 16.7% and 95.2%. Consequently, we confine ourselves to those ranges in reporting the Portuguese ethnic- or immigrant density specific marginal effects obtained from the interaction model. 

A total of 21.6% of the students in our sample reported being current smokers. With 20.3%, this number was slightly lower among students of Portuguese descent. Among native Luxembourgish students 22.1% reported being current smokers. As can be seen from Table 2 the ICC on the class level for the null model was 18% and the ICC on the school-level was 3%. Mean age in class, relative age, educational track, time spent with friends after 8 p.m., and disliking school all had highly significant detrimental effects. 

#### 3.2.1. Portuguese Ethnic Density

**Figure 2:**
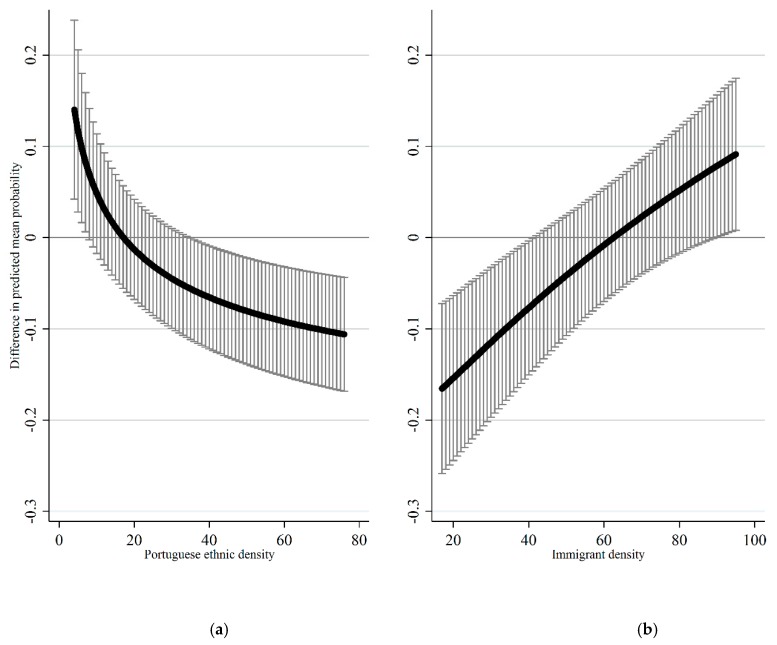
(**a**) Average marginal effect of being of Portuguese descent by Portuguese ethnic density in school-class with 95% confidence intervals; (**b**) average marginal effect of being of Portuguese descent by immigrant density in school-class with 95% confidence intervals. Reference category: Native. Note all other variables are held at their observed values in the data.

Inspection of the cross-level interaction term between being of Portuguese descent and Portuguese ethnic density in Table 2 reveals that differences in smoking behavior between students of Portuguese descent and their native classmates differ significantly based on the share of Portuguese co-ethnics in class. Figure 2 details the average marginal effect of being of Portuguese descent (i.e., the difference between the predicted probability of being a current smoker for adolescents of Portuguese descent and the predicted probability of being a current smoker for native adolescents) for all levels of Portuguese ethnic density observed in the data. Inspection of average marginal effects reveal that the effect of being of Portuguese descent switches direction based on levels of Portuguese ethnic density. Being of Portuguese descent increases the likelihood of being a smoker if the Portuguese ethnic density in class is 9% or lower, but if the Portuguese ethnic density is 31% or higher being of Portuguese descent decreases the likelihood of being a smoker.

#### 3.2.2. Immigrant Density

We can see from the cross-level interaction term between being of Portuguese descent and immigrant density in Table 2 that differences in smoking behavior between students of Portuguese descent and their native classmates differ significantly based on the share of immigrants in class. Figure 2 details the average marginal effect of being of Portuguese descent for all levels of immigrant density observed in the data. At low to medium levels up until around 45% immigrant density, being of Portuguese descent decreases the likelihood of current smoking. At high levels starting from 83%, the direction of the effect is the opposite with being of Portuguese descent increasing the likelihood of smoking. 

## 4. Discussion

In the current study we investigated the effects of school-class ethnic composition on current smoking among immigrant adolescents. We distinguished two different factors of school-class ethnic composition: co-ethnic density and immigrant density and assessed them simultaneously to disentangle the individual contribution of each factor. In a first step, we investigated the direct effect of both factors on current smoking among the subsample of students of Portuguese descent and found that both exert significant effects on current smoking. These findings are in line with other studies, testifying to the role that peers play in adolescent substance use, in general, and smoking, in particular [25,41]. In line with our hypotheses, both factors exerted opposing effects with co-ethnic density being protective of and immigrant density being conducive to current smoking. These findings testify to the distinctiveness of both factors and highlight the importance of assessing them simultaneously. The observed protective effect of co-ethnic density is in line with similar results from previous studies assessing the effect of school- or neighborhood level co-ethnic density on substance use and other health behavioral outcomes [16,17,42]. Following our initial theoretical deliberations, the underlying reasons for this protective effect might be decreased levels of acculturative stress and increased levels of social support among the students of Portuguese descent in classes with more co-ethnics. Another possible reason might lie in culture specific substance use norms. Transnationalism theory suggests that substance use norms among first- as well as second-generation immigrants might still be informed by those prevalent in their country of origin [43]. In support of this hypothesis, some studies found that less widespread alcohol use in the country of origin was associated with less alcohol consumption among immigrant adolescents [43,44]. Similarly, the lower prevalence of current smoking among adolescents of Portuguese descent in Luxembourg when compared to native Luxembourgish adolescents might be linked to the lower prevalence of smoking among adolescents in Portugal when compared to their counterparts in Luxembourg [24]. For students of Portuguese descent attending classes with many co-ethnics the protective effect of these more restrictive substance use norms might be even more pronounced as high levels of co-ethnic density might create a social environment in which these normative orientations are continuously validated and therefore perpetuated [45]. 

The observed detrimental effect of immigrant density, stands in contrast to previous findings showing a protective effect of immigrant density on similar outcomes [20,21]. However, since those studies did not include co-ethnic density in their model as a potential confounder, the protective effect they observed cannot clearly be attributed to immigrant density as such. We cannot, in the face of potentially high correlations between both factors, rule out the possibility that immigrant density has merely picked up the effect of co-ethnic density in those studies. Following our initial theoretical deliberations, the underlying reasons for this detrimental effect might lie in increased interethnic conflict and identity threat caused by a large share of immigrants in class. 

In a second step, using the whole sample, we assessed whether the effect of being of Portuguese descent differed based on the ethnic composition of the school-class. We found that while students of Portuguese descent in classes with few other students of Portuguese descent were more likely to be current smokers than their native Luxembourgish classmates, in classes with many students of Portuguese descent the opposite was the case. In contrast, in classes with few immigrants in total, students of Portuguese descent were less likely to smoke than their native Luxembourgish classmates and in classes with many immigrants, they were more likely to smoke. This suggests that differences in substance use behavior between certain ethnic minority groups and the native population might be conditional upon a reinforcing social environment. Looking at it from another perspective, the fact that there were significant interactions between being of Portuguese descent, as opposed to native Luxembourgish, and both measures of school-class ethnic composition also means that adolescents of Portuguese descent are differently affected by those factors than their native classmates. This finding corroborates our theoretical deliberations and helps rule out the possibility of confounding through unobserved class-level characteristics. 

There are some limitations to this study. One limitation lies in our operationalization of being of Portuguese descent, which we assessed based on adolescents’ own and their parents’ country of birth. Given that many of the proposed mechanisms underlying the protective effect of co-ethnic density presuppose a certain cultural similarity and shared group-identity between co-ethnics, it arguably would have been preferable to assess ethnicity based on ethnic self-identification. Due to a lack of data availability, we were unable to do this. Another limitation lies in the fact that we were unable to assess whether the observed detrimental effect of immigrant density on students of Portuguese descent was due to increased exposure to very specific ethnic groups. This might be the case if, as suggested earlier, identity threat was a major contributor to this effect. Identity threat theory [46], posits that it is particularly the presence of similar out-groups that leads to unfavorable out-group attitudes and, consequently, inter-ethnic conflict, among members of minority ethnic groups. Similarly, and in line with the reasoning of Veldhuizen and colleagues [19], it might be particularly increased exposure to very specific minority ethnic groups that causes the detrimental effect on the students of Portuguese descent in our sample. Unfortunately, the co-ethnic density for none of the other minority ethnic groups in our sample had a wide enough range to investigate those effects.

Perhaps most importantly, the case of adolescents of Portuguese descent in Luxembourg is a very specific one. Luxembourg has the highest proportion of immigrants of all European countries, and immigrants of Portuguese descent are, by far, the largest group among them. While this provided us with a wide enough range in both co-ethnic and immigrant density in school-classes to investigate both factors simultaneously and disentangle their respective effects, it also limits the generalizability of our results to other immigrant groups in other, especially less diverse, national contexts. Being able to replicate our analyses for other ethnic groups in other national contexts would have also helped us shed some more light on underlying mechanisms. If the protective effect of co-ethnic density, for instance, is specific to adolescents of Portuguese descent in Luxembourg it might be attributable to cultural differences in substance use norms that become perpetuated or mitigated depending on the ethnic composition of one’s immediate social environment. If, however, the same protective effect of co-ethnic density would be found for other ethnic groups in other national contexts, this would be an indication that increases in social support or decreases in acculturative stress play a major role in it. Further research explicitly modeling different pathways is needed to understand the underlying mechanisms causing the observed effects. 

## 5. Conclusions

The current study suggests that school-class ethnic composition might be a contributing factor to immigrant adolescent substance use. It furthermore highlights the importance of distinguishing between the share of co-ethnics in class and the overall share of immigrants in class as both might affect substance use among immigrant adolescents in contrasting ways. Given that high levels of immigrant density might go hand in hand with high levels of co-ethnic density at least for the largest ethnic groups, this means that researchers interested in assessing the effect of one of those factors need to be aware of potential confounding through the other. 

We argued that the observed protective effect of co-ethnic density might be attributable to increased levels of social support and decreased levels of acculturative stress and that the observed detrimental effect of immigrant density might be a result of increased levels of inter-ethnic conflict or identity threat. Since testing these mechanisms falls beyond the confines of this study, further research is needed to examine these conjectures. 

The current study, furthermore, indicates that differences in substance use behavior between adolescents who are part of certain ethnic minority groups and native adolescents might be contingent upon the ethnic composition of their social environment. As such, researchers interested in inter-ethnic differences in substance use should consider the ethnic composition of one or multiple of their participants’ most important social environments when investigating such differences. 

## Figures and Tables

**Table 1:** Analyses for the subgroup of students of Portuguese descent: Direct effects of co-ethnic and immigrant density.

Variable	Odds Ratio (95% Confidence Interval)
Log (% Co-Ethnic Density)	0.60 (0.39–0.92) *
Log (% Immigrant Density)	3.89 (1.61–9.41) **
Mean Age in Class	1.47 (1.31–1.65) ***
Relative Age ^a^	1.19 (0.93–1.52)
Gender (Reference = Male)	1.42 (1.04–1.96) *
Socio-Economic Status	0.98 (0.91–1.06)
First Generation	1.07 (0.72–1.59)
Luxembourgish Spoken at Home	1.53 (1.06–2.19) *
Portuguese Spoken at Home	1.46 (0.73–2.9)
Disliking School	1.52 (1.24–1.88) ***
Meet Friends after 8 p.m.	1.4 (1.2–1.63) ***
Educational Track (Reference = High)	
Middle	1.82 (1.06–3.11) *
Low	2.71 (1.29–5.68) **
Intraclass Correlation (Class) ^b^	0.16
Var. random intercept ^b,c^	0.62 (0.32–1.19) **

^a^ Centered around the class mean, ^b^ For the null model, ^c^ One-tailed adjusted Wald test, * *p* < 0.5; ** *p* < 0.01; *** *p* < 0.001 (two-tailed test).

**Table 2:** Analyses for the whole sample: Interaction between being of Portuguese descent and co-ethnic and immigrant density.

Variable	Odds Ratio (95% Confidence Interval)
Portuguese X Log (% Portuguese Ethnic Density)	0.54 (0.41–0.72) ***
Portuguese X Log (% Immigrant Density)	4.07 (1.58–10.48) **
Mean Age in Class	1.42 (1.33–1.52) ***
Relative Age ^a^	1.42 (1.27–1.59) ***
Gender (Reference = Male)	1.16 (0.95–1.41)
Socio-Economic Status	1 (0.96–1.04)
First Generation	1.05 (0.78–1.42)
Country of Origin (Reference = Native)	
Portuguese	0.017 (0–0.65) *
Other	0.38 (0.05–2.89)
Disliking School	1.57 (1.41–1.75) ***
Meet Friends after 8 p.m.	1.62 (1.48–1.79) ***
Luxembourgish Spoken at Home	1.28 (0.99–1.65)
Portuguese Spoken at Home	1.11 (0.74–1.68)
Educational Track (Reference = High)	
Middle	2.18 (1.67–2.85) ***
Low	3.11 (2.16–4.48) ***
Log (% Portuguese Ethnic Density)	1.01 (0.86–1.18)
Log (% Immigrant Density)	1.03 (0.71–1.5)
Other X Log (% Portuguese Ethnic Density)	0.87 (0.73–1.05)
Other X Log (% Immigrant Density)	1.4 (0.78–2.5)
Intraclass Correlation (Class) ^b^	0.18
Var. random intercept (Class) ^b,d^	0.59 (0.44–0.78) ***
Var. random slope Portuguese (Class) ^c,d^	0.13 (0–4.35)
Intraclass Correlation (School) ^b^	0.03
Var. random intercept (School) ^b,d^	0.11 (0.05–0.29) *

^a^ Centered around the class mean, ^b^ For the null model, ^c^ For the model omitting immigrant- and Portuguese ethnic density and their cross-level interactions, ^d^ One-tailed adjusted Wald test, * *p* < 0.5; ** *p* < 0.01; *** *p* < 0.001 (two-tailed test).
